# *Coxiella burnetii* Whole Cell Vaccine Produces a Th1 Delayed-Type Hypersensitivity Response in a Novel Sensitized Mouse Model

**DOI:** 10.3389/fimmu.2021.754712

**Published:** 2021-09-20

**Authors:** Alycia P. Fratzke, Anthony E. Gregory, Erin J. van Schaik, James E. Samuel

**Affiliations:** ^1^Department of Veterinary Pathobiology, College of Veterinary Medicine and Biomedical Sciences, Texas A&M University, College Station, TX, United States; ^2^Department of Microbial Pathogenesis and Immunology, College of Medicine, Texas A&M University, Bryan, TX, United States; ^3^Department of Physiology & Biophysics, School of Medicine, University of California Irvine, Irvine, CA, United States

**Keywords:** *Coxiella burnetii*, delayed-type hypersensitivity, mouse model, Q fever, vaccine, Th1

## Abstract

Q-VAX®, a whole cell, formalin-inactivated vaccine, is the only vaccine licensed for human use to protect against *Coxiella burnetii*, the cause of Q fever. Although this vaccine provides long-term protection, local and systemic reactogenic responses are common in previously sensitized individuals which prevents its use outside of Australia. Despite the importance of preventing these adverse reactions to develop widely accepted, novel vaccines against *C. burnetii*, little is understood about the underlying cellular mechanisms. This is mostly attributed to the use of a guinea pig reactogenicity model where complex cellular analysis is limited. To address this, we compared three different mouse strains develop a model of *C. burnetii* whole cell vaccine reactogenic responses. SKH1 and C57Bl/6, but not BALBc mice, develop local granulomatous reactions after either infection- or vaccine-induced sensitization. We evaluated local and systemic responses by measuring T cell populations from the vaccination site and spleen during elicitation using flow cytometry. Local reaction sites showed influx of IFNγ+ and IL17a+ CD4 T cells in sensitized mice compared with controls and a reduction in IL4+ CD4 T cells. Additionally, sensitized mice showed a systemic response to elicitation by an increase in IFNγ+ and IL17a+ CD4 T cells in the spleen. These results indicate that local and systemic *C. burnetii* reactogenic responses are consistent with a Th1 delayed-type hypersensitivity. Our experiments provide insights into the pathophysiology of *C. burnetii* whole cell vaccine reactogenicity and demonstrate that C57Bl/6 and SKH1 mice can provide a valuable model for evaluating the reactogenicity of novel *C. burnetii* vaccine candidates.

## Introduction

*Coxiella burnetii* is a facultative intracellular, Gram-negative bacterium and the cause of the zoonotic disease Q fever. Acute Q fever is typically self-limiting causing fever, headache, and myalgia. However, severe infections may cause atypical pneumonia, hepatitis, myocarditis, and spontaneous abortion (1–4). Approximately 1 to 2% of patients with clinical symptoms develop chronic syndromes such as Q fever fatigue syndrome or valvular endocarditis (1, 5, 6). Infection in humans is usually due to exposure to reservoir species such as sheep, goats, cattle, and camels, or due to contact with contaminated animal products (1, 7, 8). Additionally, the Centers for Disease Control and Prevention (CDC) has designated *C. burnetii* as a select agent and potential weapon for bioterrorism because of its low infectious dose, persistence in the environment, and aerosol transmission (6). Q-VAX^®^ (Seqirus), a formalin-inactivated, whole cell vaccine (WCV) for *C. burnetii* is licensed for use in humans only in Australia (9, 10). Although this vaccine provides long-term protection against Q fever, severe local and systemic reactions to Q-VAX in sensitized individuals have prevented the licensure of this vaccine elsewhere (11, 12). To reduce the rate of vaccine reactions, individuals must undergo costly, time-consuming pre-vaccination screening including anti-*C. burnetii* titers and intradermal skin testing (9). This risk of adverse reactions with the whole cell *C. burnetii* vaccine is a major barrier to the availability of a protective vaccine worldwide not only for occupationally at-risk populations, but also military services seeking protection against a possible bioterrorism agent.

Despite decades of research to develop novel vaccine strategies against *C. burnetii*, the pathophysiology and causes of the adverse reactions to the current vaccine are poorly understood. Early researchers postulated that the phase I lipopolysaccharide (LPS) of *C. burnetii* was the cause of WCV reactogenicity, but recent reports show that this is not true (13–16). Although little information has been published on the mechanisms underlying *C. burnetii* WCV reactions, clinical and histopathologic evaluations provide some insights. *C. burnetii* WCV reactions have a delayed onset and are more common and more severe in individuals with prior sensitization (9–11). This suggests that *C. burnetii* WCV reactogenic responses are a type IV hypersensitivity reaction. Type IV hypersensitivity, also known as delayed-type hypersensitivity, is caused by memory T cells which produce Th1-type cytokines, such as interferon γ (IFNγ), IL2, and tumor necrosis factor β (TNFβ), or Th2-type cytokines, such as IL4, IL5, and IL13 (17–19).

Type IV hypersensitivity is also subdivided into contact, tuberculin, and granulomatous types. Contact hypersensitivity is caused when haptens bind to host proteins to form new antigens that are taken up by Langerhans cells and then stimulate T cell responses. Tuberculin and granulomatous hypersensitivities occur when antigens that penetrate tissues are taken up by dendritic cells which then present to and activate T cells. Tuberculin and contact hypersensitivities occur at 48-72 hours after exposure, while granulomatous hypersensitivity has a delayed onset, with an average of 21-28 days. Granulomatous hypersensitivity is also characterized by a marked influx of activated macrophages caused by antigens which are difficult to digest (17). Histopathology of local *C. burnetii* WCV reactions in humans and guinea pigs show granulomatous inflammation characterized by an influx of epithelioid macrophages, lymphocytes, and neutrophils with formation of abscesses. In guinea pigs, local granulomas are most severe at 12-15 days post-exposure and, in humans, these reactions have been reported to last several weeks to a few years (11, 20). Together this suggests that *C. burnetii* WCV reactogenic responses are a granulomatous type IV hypersensitivity. However, this hypothesis is unproven and many questions remain, such as how T cells mediate local and systemic reactions during elicitation, how these pathologic adaptive responses develop during sensitization, and what is the inciting cause of these reactions.

Understanding the differences between protective and pathologic responses to vaccination is essential to develop safe, novel vaccines against *C. burnetii* and other infectious agents. Currently, the standard model for studying WCV reactogenicity is a sensitized guinea pig model (16, 21). Guinea pigs are highly susceptible to *C. burnetii* infection, develop pulmonary lesions after intratracheal infection similar to those described in humans, and readily develop reactions to WCV. However, the lack of immune markers for this species severely inhibits in-depth investigation of local and systemic immune responses (11, 16, 22). Therefore, we developed a novel mouse model of the *C. burnetii* WCV reactogenic response for use in immunologic studies of adverse vaccine reactions. We then determined the elicitation dose that maximizes lesions in sensitized compared to unsensitized animals to target adaptive responses. Finally, we show that WCV reactions contain IFNγ- and IL17a-producing CD4 T cells. Our work demonstrates that reactions to *C. burnetii* whole cell vaccine are a Th1-mediated type IV hypersensitivity.

## Materials and Methods

### Bacterial Strains and Vaccine Materials

For intratracheal infections, *C. burnetii* Nine Mile phase I (NMI) clone 7 (RSA493) was grown in embryonated yolk sacs, then purified using gradient centrifugation as described previously (22). To produce WCV, cultures of *C. burnetii* NMI RSA493 were grown in ACCM-2 media as described in Omsland et al. (23), then inactivated in 2% formalin for 48 hours (14, 23). WCV was administered as 2 µg, 10 µg, 30 µg, or 50 µg doses by dry weight (1 mg WCV = 3.7x10^10^ cells) (24). Experiments involving live *C. burnetii* NMI RSA493 were performed in biosafety level 3 (BSL3) facilities at Texas A&M Health Science Center.

### Experimental Animals

Female C57Bl/6NHsd (C57) and BALB/c mice (BAL), 6-8 weeks old, were purchased from Envigo (Huntingdon, UK) and female SKH1-Elite (SK) mice, 6-8 weeks old, were purchased from Charles River Laboratories (Wilmington, MA). Mice were housed in microisolator cages under pathogen-free conditions and given free access to food and water. Animals were housed in approved animal biosafety level 3 or level 2 facilities and all experiments were performed under an animal use protocol approved by the Institutional Animal Care and Use Committee at Texas A&M University.

### Sensitization and Elicitation of Responses

For infection-sensitization (NMI), mice were intratracheally inoculated with 10^5^ or 10^6^ genomic equivalents (GE) of live *C. burnetii* as previously described with some modifications (22). Briefly, mice were anesthetized by intraperitoneal injection of 100 mg/kg ketamine and 10 mg/kg xylazine. Mice were then placed on a Mouse Intubation Platform (Penn-Century; Wyndmoor, USA) at a 45° angle and a 20-gauge catheter, needle removed, was inserted into the trachea. Live bacteria were administered through the catheter in 30 µL of sterile PBS. Infection-sensitized mice were monitored for clinical signs and weighed three times per week for two weeks post-inoculation. For vaccine-sensitization (WCV), mice were anesthetized as above and 50 µg of WCV in 50 µL of sterile PBS was administered subcutaneously (SC) in the middle of the back. Unsensitized control mice (PBS) were given a subcutaneous vaccination with 50 µL PBS alone. Mice were rested for 5-6 weeks post-sensitization prior to elicitation.

For elicitation of vaccine reactions mice were anesthetized as above. In haired mice, vaccination sites were first shaved using electric clippers followed by the application of a depilatory cream for 30 seconds. Mice were vaccinated SC with 2 µg, 10 µg, or 30 µg of WCV in 50 µL of sterile PBS or with 50 µL sterile PBS alone into the right and left flanks. Vaccine sites were visually monitored daily for two weeks and local induration was measured using calipers, then mice were euthanized and tissues were collected for histopathology or flow cytometry.

### Serum Antibody Responses

Serum samples were collected from either the submandibular vein or by intra-cardiac stick at pre-sensitization, post-sensitization, and post-elicitation time points for measurement of anti-*C. burnetii* IgG titers. Briefly, flat-bottomed 96-well plates were coated in 5 µg/mL *C. burnetii* antigen overnight then blocked in 3% powdered milk for 2 hours. Serum was pooled from 5 mice per group, diluted in PBS with 1% powdered milk, and serial dilutions were applied to the plate and incubated for 2 hours at 37°C. After washing three times with PBS with 0.05% tween 20, plates were then incubated with HRP-conjugated goat anti-mouse IgG antibody (1:10,000) for 2 hours at 37°C. 3,3′,5,5′-Tetramethylbenzidine (TMB) was used as substrate and OD was measured at 490 nm by a Biotek 800 TS Absorbance Reader.

### Histopathology and Immunohistochemistry

Vaccination sites, lungs, and spleens were collected into 10% neutral buffered formalin and fixed for a minimum of 48 hours. Tissues were serially trimmed (2-4 sections per tissue) and placed in cassettes before submission to AML Laboratories (Jacksonville, FL, USA) for processing, embedding, and sectioning at 5 µm. Slides were stained with hematoxylin and eosin (HE). Histopathology of vaccine sites was assessed on de-identified, HE-stained slides using semi-quantitative scoring by a board-certified veterinary pathologist. Vaccine sites were scored from 0-5 based on lesion size, immune cell infiltrate, and areas of suppurative necrosis. Briefly, 0: no lesions, 1: minimal immune cell infiltrate, 2: mild, focal immune cell infiltrate, 3: moderate multifocal immune cell infiltrate, 4: moderate to severe, diffuse immune cell infiltrate, 5: severe, diffuse immune cell infiltrate with areas of suppurative necrosis.

For immunohistochemistry (IHC), unstained slides were deparaffinized and rehydrated by incubating in three washes of xylene, three washes of 100% ethanol, and one wash in 95% ethanol, 70% ethanol, 50% ethanol, and deionized water for 3 minutes each. Slides were then incubated in 3% hydrogen peroxide at room temperature for 10 minutes. Antigen retrieval was performed by incubating slides in Tris-EDTA buffer (10mM Tris Base, 1 mM EDTA, 0.05% Tween 20, pH 9.0) at 100°C for 20 min followed by washing in tap water for 10 min. Slides were blocked in TBS with 1% powdered milk for 2 hours. Primary antibodies were diluted in TBS with 0.5% bovine serum albumin (BSA) and applied to slides for 2 hours at room temp or overnight at 4°C. Slides were washed in TBS with 0.025% Triton-X 100 then incubated with HRP-conjugated goat anti-rabbit IgG diluted in TBS with 0.5% BSA for 2 hours at room temp or overnight. For fluorescence, Tyramide Reagents (Thermofisher) were applied to the slide per manufacturer directions for 10 min at room temp, then antigen retrieval was repeated for 2 min to strip antibodies before repeating the antibodies for the next antigen. Finally, TrueVIEW Autofluorescence Quenching Kit with DAPI (Vector, cat. SP-8500) was used to decrease autofluorescence, stain nuclei with DAPI and coverslip the slides. Slides were allowed to cure overnight at room temp.

HE-stained and IHC slides were scanned at 20X magnification using an Olympus VS120 Slide Scanner (Integrated Microscopy and Imaging Laboratory, Texas A&M University). For HE slides, brightfield images were collected. For fluorescent slides, DAPI, FITC, and TxRed channels were used to acquire images. Lesion measurements and cell counts on slide images were performed using QuPath v0.2.0-m8 (25). Neutrophils, macrophages, and lymphocytes were evaluated by morphology and quantified by counting cells within ten representative 100 µm^2^ fields on H&E. For CD3+ T cells and CD19+ B cells, all cells within the lesions area were quantified using positive cell analysis on IHC slides.

Primary antibodies were purchased from Cell Signaling Technology: anti-CD3ϵ (D7A6E) and anti-CD19 (D4V4B). HRP-conjugated Goat anti-rabbit IgG (Novus Biologicals) diluted 1:1000 was used as the secondary antibody. Fluorescent Tyramide Regents (Thermofisher) used were AlexaFluor 488 and AlexaFluor 555 or AlexaFluor 594.

### Flow Cytometry

For spleens, single cell suspensions were produced by pressing spleens through a 70 µm cell strainer in cold 3 mL FACs buffer (PBS with 2% fetal bovine serum, 0.1% NaN3, pH 7.2) then washing with FACs buffer. Splenocytes were centrifuged then resuspended in ACK Lysing Buffer (Thermofisher) for 1 min to remove red blood cells. Cells were resuspended in 1 mL RPMI complete and an aliquot was mixed with 10 µL trypan blue to quantify cells using a Countess II (Thermofisher). Splenocytes were then diluted to 10^7^ cells/mL.

For vaccination sites, a 10 mm punch biopsy was used to collect the skin and subcutis at the elicitation site. Using a razor blade the subcutis and dermis were scraped from the overlying epidermis and placed in a gentleMACS C-tube for processing using the gentleMACS mouse adipose tissue dissociation kit (Miltenyi Biotec). Vaccine sites were dissociated using the Miltenyi Biotec gentleMACS Octo Dissociator with heaters. Cell suspensions were centrifuged and resuspended in 1 mL RPMI complete and cells were quantified as above then diluted to 10^7^ cells/mL.

Cells were centrifuged and resuspended in 1:1000 anti-mouse CD16/CD32 in FACs buffer and incubated on ice for 10 min. Next cells were stained with 1 µL/mL Zombie Violet or Zombie Aqua live/dead dye (Biolegend) and incubated on ice for 5 min. Cells were then incubated in fluorochrome-conjugated cell surface antibodies on ice for 30 min. For intracellular staining, cells were fixed and permeabilized using FoxP3/Transcription Factor Staining Buffer Set (eBioscience) followed by incubation with intracellular antibodies for 30 min on ice. Cells were then resuspended in FACs buffer and kept at 4°C until analysis. For intracellular cytokine and FoxP3 expression, cell suspensions from spleens and vaccine sites were aliquoted into a round-bottomed 96-well plate with or without 20 µg/mL WCV and cultured for 18 hours at 37°C with 5% CO_2_ followed by the addition of GolgiPlug (BD Biosciences) for 6 hours prior to antibody staining. Stimulation of cells by 50ng/mL of phorbol 12-myristate 13-acetate (PMA) and 1mM ionomycin for 6 hours was used as a positive control (Data not shown).

Flow cytometric antibodies included CD3ϵ-PE/Cy7, CD8-FITC, CD4-APC/Cy7, IL17a-BV711, IFNγ-APC, IL4-PE, FoxP3-BV421, CD69-BV711, CD44-PE, and CD62L-APC (BioLegend). Cells were counted using the BD LSRFortessa X-20 Flow Cytometer and analyzed using FlowJo v10.6.2 (FlowJo LLC.).

### Statistics

Statistical analyses were calculated using Prism v7.0 (GraphPad Software Inc.). Results were compared using one-way ANOVA with Dunnett’s correction for multiple comparisons. Differences were considered significant if p-value ≤ 0.05 (*), ≤ 0.01 (**), ≤ 0.001 (***), or ≤ 0.0001 (****).

## Results

### Sensitized Mice Produce Local Reactogenic Responses to *C. burnetii* WCV

**Figure 1:**
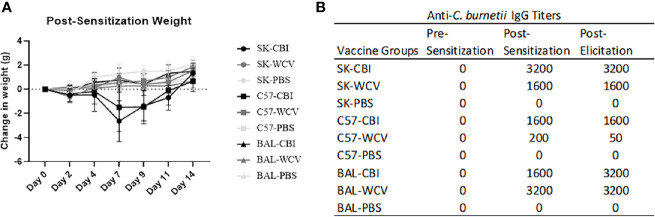
Weight and antigen-specific IgG responses to sensitization methods. **(A)** Weight changes during 14 days post-sensitization. Infection-sensitized SK and C57 show transient weight loss. The graph shows the means of each group with error bars that represent the standard error of the mean, n=5 mice per group. **(B)** Anti-*C. burnetii* IgG responses to infection-, vaccine-, and sham-sensitized mice. Infection- and vaccine-sensitization produced similar IgG titers in SK and BAL mice. Vaccine-sensitized C57 mice produced reduced IgG response compared to infection-sensitized. Serum IgG results from pooled sera (n=5) represent values one standard deviation above the mean of negative control sera.

Guinea pigs are the current animal model of choice for evaluation of hyper-reactive lesions to *C. burnetii* vaccines, however, antibodies targeting guinea pig cell markers are limited which inhibits immunologic investigation of these responses. To address this, we developed a novel mouse model of the *C. burnetii* whole cell vaccine reactogenic response. To develop our model, we tested the ability to reproduce *C. burnetii* reactogenic responses in SKH1(SK), C57Bl/6 (C57), and BALBc (BAL) mice. SK mice are outbred, immune-competent, and hairless due to a mutation in the *Hr* gene (26). C57 and BAL mice were chosen as Th1- and Th2-biased strains, respectively, to compare responses by immunophenotype (27). C57, BAL, and SK mice (n=4-5/group) were sensitized to *C. burnetii* by intratracheal inoculation with 10^5^ or 10^6^ GE *C. burnetii* RSA493 (NMI), by SC vaccination with 50 µg WCV in 50 µL sterile PBS (WCV), or by SC vaccination with 50 µL sterile PBS (PBS) (22). Mice were monitored for 14 days post-sensitization by measuring weight change. Infection-sensitized SK and C57 mice showed transient weight loss followed by recovery by day 14 while infection-sensitized BAL, all vaccination-sensitized, and all control groups showed no overt weight loss over the observation period (**Figure 1A**). For elicitation, mice were given a SC vaccination of 10 µg WCV in 50 µL sterile PBS and PBS alone into the right and left flanks, respectively, and vaccination sites were monitored for swelling and erythema daily for 14 days until euthanasia. Day 14 post-elicitation was chosen as the endpoint based on previous reports and our own work in guinea pigs showing induration is most severe at approximately 11-15 days post-vaccination (11). Mice in all experimental groups showed no evidence of weight loss during elicitation (**Supplementary Figure 1**).

Blood samples were collected prior to sensitization (pre-sensitization), one day before elicitation (post-sensitization), and at necropsy (post-elicitation) to evaluate antigen-specific antibody formation in serum as a measure of sensitization. Anti-*C. burnetii* IgG titers showed seroconversion at post-sensitization and post-elicitation time points in both infection- and vaccine-sensitized groups in all mouse strains. SK and BAL mice as well as infection-sensitized C57 mice produced IgG titers of ≥1:1600. Vaccine-sensitized C57 mice produced modest IgG titers of 1:200. No anti-*C. burnetii* IgG titers were detected in unsensitized controls at any time point (**Figure 1B**).

**Figure 2:**
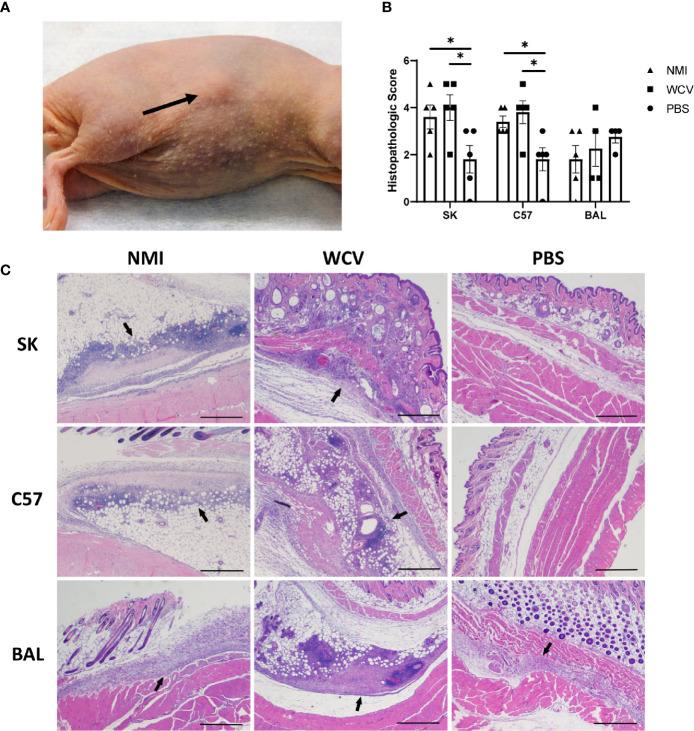
Responses to WCV in sensitized and unsensitized mice. **(A)** Example of the local induration observed in SK mice, day 14 post-elicitation (arrow). **(B)** Histopathologic scores of local vaccination sites based on lesion size, immune cell infiltrate, and areas of suppurative necrosis. Lesions are more severe in infection- and vaccine-sensitized C57 and SK mice. **(C)** Representative histopathology of local vaccination sites. Marked local inflammation (arrow) is evident in infection- and vaccine- sensitized SK and C57 mice. BAL mice show variable inflammation with either sensitization method. 2x, HE stain, bar= 500 µm. Graphs show the means of each group with error bars that represent the standard error of the mean, n=4-5 mice per group. Data were analyzed using one-way ANOVA with Dunnett’s correction for multiple comparisons. Asterisks indicate significant differences between groups (*p < 0.05).

Focal swellings at the WCV site were observed beginning at day 8 in infection-sensitized and day 10 in vaccine-sensitized SK mice, but only occurred in 3 of 5 mice in each of these groups (**Figure 2A** and **Supplementary Figure 1**). The vaccine site swellings measured 2 mm thick using skin calipers compared to 1 mm in normal mouse skin. Swellings were not observed grossly in the control SK mice or any of the C57 and BAL mouse groups, however, hair regrowth in both strains and skin pigmentation in C57 mice were common during the elicitation period.

Vaccination sites were collected in formalin at 14 days post-elicitation for histopathology. HE-stained slides of vaccination sites showed more severe lesions in infection- and vaccine-sensitized C57 and SK mice compared to unsensitized controls, but vaccine lesions in BAL mouse groups did not significantly differ (**Figure 2B**). Local reactive lesions consisted of infiltrates of macrophages, neutrophils, and lymphocytes with multifocal areas of suppurative necrosis and degeneration (**Figure 2C**). Infection- and vaccine-sensitized lesions in C57 and SK mice did not significantly differ by the type of cellular infiltrate or overall severity of lesions.

### Severity of Local WCV Reactions Is Dose-Dependent

**Figure 3:**
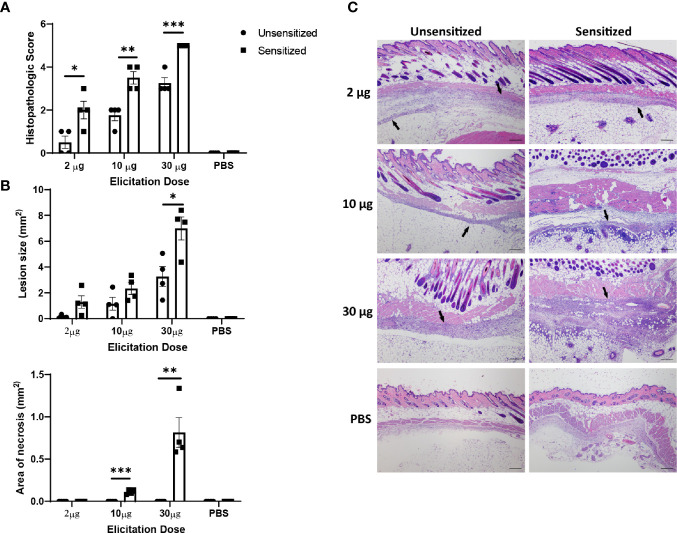
Severity of vaccine site reactions to (*C*) *burnetii* WCV are dose-dependent. **(A)** Histopathologic scores of local vaccination sites in C57 mice based on lesion size, immune cell infiltrate, and areas of suppurative necrosis. Reactions are significantly more severe in sensitized mice with at all doses evaluated. Local reactions are dose-dependent in both sensitized and unsensitized mice. **(B)** Lesion size and area of necrosis in local reaction sites. Only 30 µg dose showed significantly greater lesion size when comparing sensitized to unsensitized mice. Necrosis was only evident in sensitized mice at 10 µg and 30 µg doses. **(C)** Representative histopathology sections from each dose group showing dose-dependent amounts of inflammatory infiltrate (arrows). 4x, HE stain, bar= 200 µm. Graphs show the means of each group with error bars that represent the standard error of the mean, n=4 mice per group. Data were analyzed using one-way ANOVA with Dunnett’s correction for multiple comparisons. Asterisks indicate significant differences between groups (*p < 0.05, **p < 0.01, ***p < 0.001).

**Figure 4:**
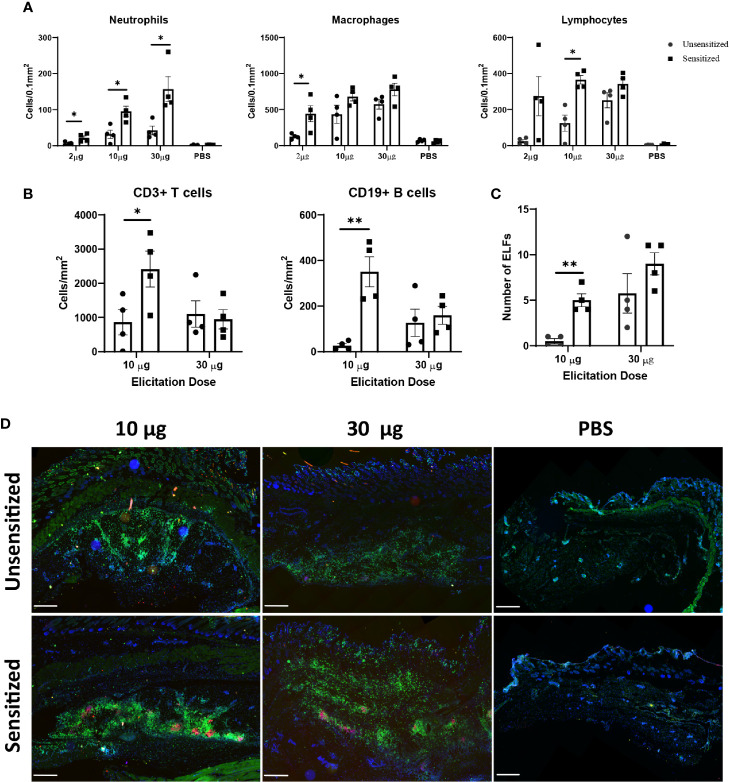
Immune cell infiltrate and ectopic lymphoid follicle (ELF) formation depend on both sensitization status and elicitation dose. **(A)** Numbers of cells within a 0.1 mm^2^ area of each vaccine site. Neutrophils were increased at all doses, however only the 10 µg dose showed significantly different lymphocyte numbers. Cells were defined by morphology and counted manually within ten representative 100 µm^2^ fields on HE stained slides. **(B)** Both T and B cell numbers are significantly greater in sensitized mice at the 10 µg dose, but not the 30 µg dose. Cells were counted using QuPath based on fluorescence immunohistochemistry results. **(C)** Total numbers of ELFs within vaccine reaction sites. Sensitized mice develop more ELFs at the 10 µg dose. **(D)** Representative IHC from vaccine sites of unsensitized and sensitized mice from 10 µg, 30 µg, and PBS injection sites. CD3+ T cells are diffusely distributed throughout the lesion while CD19+ B cells are confined to clusters within ectopic lymphoid follicles. 2x, anti-CD3 (green), anti-CD19 (red), nuclei (blue), bar=500 µm. Graphs show the means of each group with error bars that represent the standard error of the mean, n=4 mice per group. Data were analyzed using one-way ANOVA with Dunnett’s correction for multiple comparisons. Asterisks indicate significant differences between groups (*p < 0.05, **p < 0.01).

Although *C. burnetii* WCV reactogenicity is mainly associated with previous sensitization, higher doses of WCV can produce significant local inflammation through innate responses that may obscure the hypersensitivity response (16). To target the hypersensitivity response, we continued by assessing multiple elicitation doses to determine which dose maximized the cellular influx produced by adaptive immunity without obscuring the lesions with excessive innate responses. C57 mice were sensitized with WCV or PBS as described above then administered a SC elicitation dose of 2 µg, 10 µg, 30 µg, and sterile PBS in a volume of 50 µL into either the right or left flank. At 14 days post-elicitation, vaccine sites were collected in formalin for histopathology and immunohistochemistry. Both sensitized and unsensitized groups showed dose-dependent responses to vaccination, however sensitized mice consistently showed significantly more severe responses than unsensitized mice at the same dose based on semi-quantitative scoring of HE-stained slides (**Figures 3A, B**). However, the mean size of the lesions only significantly differed between sensitization groups at the 30 µg dose (**Figure 3C**). Interestingly, suppurative necrosis was only evident in sensitized mice at the 10 µg and 30 µg doses (**Figure 3C**). Individual cell counts of neutrophils, lymphocytes, and macrophages showed variable differences within dose groups. Neutrophils were increased in sensitized mice compared to unsensitized mice at all doses, while macrophages were only increased at the 2 µg dose and lymphocytes were only increased at the 10 µg dose when comparing sensitization (**Figure 4A**).

Immunohistochemistry was used to further differentiate lymphocytes into CD3+ T cells and CD19+ B cells. At the 10 µg dose, both B and T cells were significantly increased in sensitized mice compared to unsensitized, but there was no difference in these populations at the 30 µg dose (**Figure 4B**). In all sections, T cells were distributed evenly throughout the lesion except in deep, often perivascular, clusters of lymphocytes forming ectopic lymphoid follicles (ELF) where they formed dense aggregates with CD19+ B cells. There were significantly more ELFs in sensitized mice at the 10 µg dose but not the 30 µg dose (**Figure 4C**) compared to unsensitized mice. In contrast, B cells were only present within ELFs (**Figure 4D**).

### WCV Reactions Induce Local Influx of IFNγ+ and IL17a+ CD4+ T Cells

Because delayed-type hypersensitivities are mediated by T cells and experiments showed a marked influx of CD3+ T cells on immunohistochemistry of vaccine site lesions, we used flow cytometry to further characterize T cell subsets that are increased during WCV reactogenic responses both locally and systemically. To do so, we extracted cells from the vaccine sites and spleens of mice at 14 days post-elicitation. Vaccine-sensitized and unsensitized mice as described above were vaccinated SC at four separate sites in the right and left flank, then spleens and vaccine sites were collected at 14 days post-elicitation. The four vaccine sites from each mouse were pooled prior to cell separation for flow cytometric evaluation. The 10 µg elicitation dose was chosen for these experiments since this dose produced maximal differences in lymphocyte responses between sensitization groups. Extracted cells were stimulated with WCV and stained for surface markers and expression of IFNγ, IL4, IL17a, and FoxP3 as markers for Th1, Th2, Th17, and Treg cells, respectively. Unsensitized mice elicited with injections of PBS only were used to compare responses to normal cell populations in the skin and spleen (Sham).

**Figure 5:**
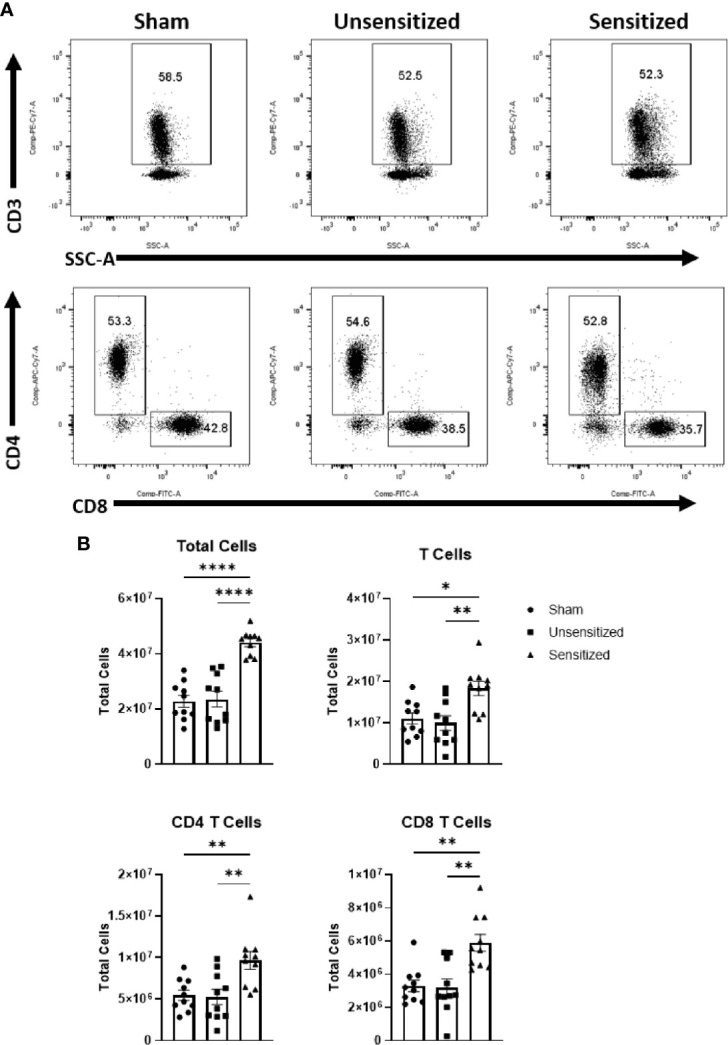
Vaccine site reactions in sensitized mice have an influx of CD4 and CD8 T cells. **(A)** Gating strategy for evaluation of T cells and subpopulations in vaccination sites. **(B)** Total cell numbers for all cells, CD3+ T cells, CD3+CD4+ T cells, and CD3+CD8+ T cells. Sensitized mice show a marked increase in all cell groups. Graphs show the means of each group with error bars that represent the standard error of the mean. Cell counts are the sum of four vaccination sites from each mouse. Data are the result of two experiments, n=10 mice per group. Data were analyzed using one-way ANOVA with Dunnett’s correction for multiple comparisons. Asterisks indicate significant differences between groups (*p < 0.05, **p < 0.01, ****p < 0.0001).

**Figure 6:**
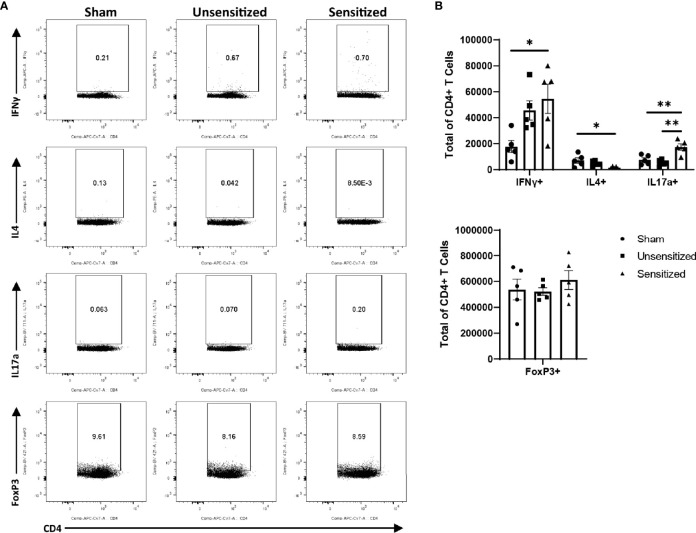
CD4 T cells within vaccine reactions in sensitized mice are IFNγ+ and IL17a+. **(A)** Representative gates for cytokine production and FoxP3 expression by CD4+ T cells. **(B)** Total CD4+ T cells expressing IFNγ, IL4, IL17a, and FoxP3 (Treg). IFNγ and IL17a expression from sensitized mice are significantly increased compared to sham while IL4 expression is significantly decreased. No differences in numbers of FoxP3+ CD4 T cells are observed between experimental groups. Graphs show the means of each group with error bars that represent the standard error of the mean. Cell counts are the sum of four vaccination sites from each mouse, n=5 mice per group. Data were analyzed using one-way ANOVA with Dunnett’s correction for multiple comparisons. Asterisks indicate significant differences between groups (*p < 0.05, **p < 0.01).

Local vaccine sites showed marked influx of total cells and T cells compared to unsensitized and sham mice (**Figures 5A, B**). CD4+ and CD8+ T cells in sensitized mice were similarly increased at vaccine sites compared to unsensitized and sham groups (**Figures 5A, B**). Evaluation of cytokine production by CD4 T cells in sensitized mice showed a significant increase in IFNγ production compared to the sham group but not unsensitized mice. Although the mean of IFNγ+ CD4+ T cells in unsensitized mice was increased compared to sham mice, this result was not significant (p=0.0818) (**Figures 6A, B**). Similarly, CD4+ IL17a-secreting cells were elevated in sensitized mice compared to unsensitized and sham mice (**Figures 6A, B**). However, unsensitized mice did not have an increase in IL17a-secretion by CD4+ T cells compared to sham mice. In contrast, IL4+ CD4+ T cells were significantly decreased in sensitized mice compared to sham (**Figures 6A, B**). FoxP3+ CD4+ T cells and production of IFNγ, IL4, and IL17a by CD8+ T cells did not significantly differ between experimental groups (**Figures 6A, B** and **Supplementary Figure 2**).

**Figure 7:**
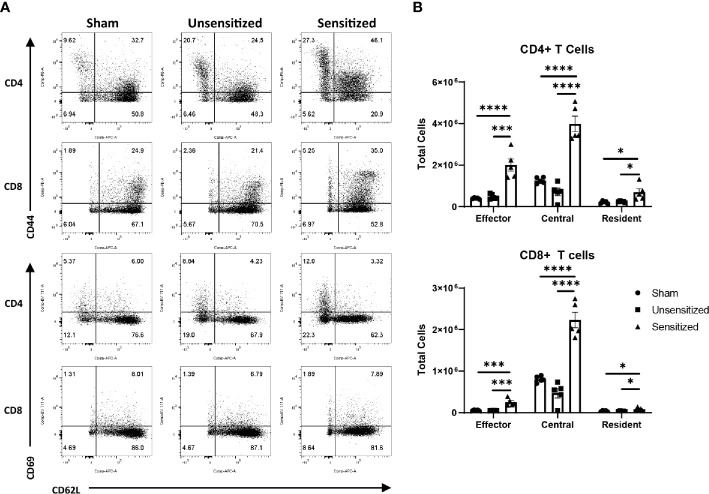
Vaccine site reactions in sensitized mice have increased of both CD4 and CD8 memory T cells. **(A)** Representative flow cytometry gates for evaluation of CD4+ and CD8+ T_EM_ (CD44^hi^, CD62L^lo^), T_CM_ (CD44^hi^, CD62L^hi^), and T_RM_ (CD44^hi^, CD69^hi^) cells from vaccine sites. **(B)** Total cell counts of memory T cells from vaccination sites. Locally, there is expansion of effector, central, and resident memory CD4 and CD8 T cells in sensitized mice compared to unsensitized and sham groups. Graphs show the means of each group with error bars that represent the standard error of the mean. Cell counts are the sum of four vaccination sites from each mouse, n=5 mice per group. Data were analyzed using one-way ANOVA with Dunnett’s correction for multiple comparisons. Asterisks indicate significant differences between groups (*p < 0.05, ***p < 0.001, ****p < 0.0001).

We next evaluated the local effector, central, and resident memory T cells populations within vaccine sites using the surface markers CD44, CD62L, and CD69. CD44 is upregulated in activated T cells and is important for recruitment to sites of inflammation while CD62L is a lymph node homing receptor (28). CD44+CD62L+ central memory T cells (T_CM_) normally circulate within the blood and lymphoid tissues and rapidly expand in response to antigen re-stimulation while CD44+CD62L- effector memory T cells (T_EM_) home to peripheral tissues in response to chemoattractants and produce cytokines in response to re-stimulation (29, 30). Similar to T_EM_, resident memory T cells (T_RM_) downregulate CD62L but also upregulate CD69 which sequesters sphingosine 1-phosphate receptor 1 (S1PR1) preventing tissue egress. This causes T_RM_ to remain within the peripheral tissues after sensitization to provide tissue-specific immune memory (29, 31). Evaluation of memory T cell populations from vaccine sites showed significant increases in CD4 and CD8 T_EM_ and T_CM_ populations in sensitized mice compared to unsensitized and sham controls (**Figures 7A, B**). CD4+ T_RM_ from sensitized mice were also significantly increased compared to unsensitized and sham controls, while CD8+ T_RM_ from sensitized mice were significantly increased compared to sham mice but not unsensitized mice. There were no significant differences in memory T cell populations between unsensitized and sham mice.

### WCV Reactions Produce Systemic Expansion of IFNγ- and IL17a-Producing CD4+ T Cells

**Figure 8:**
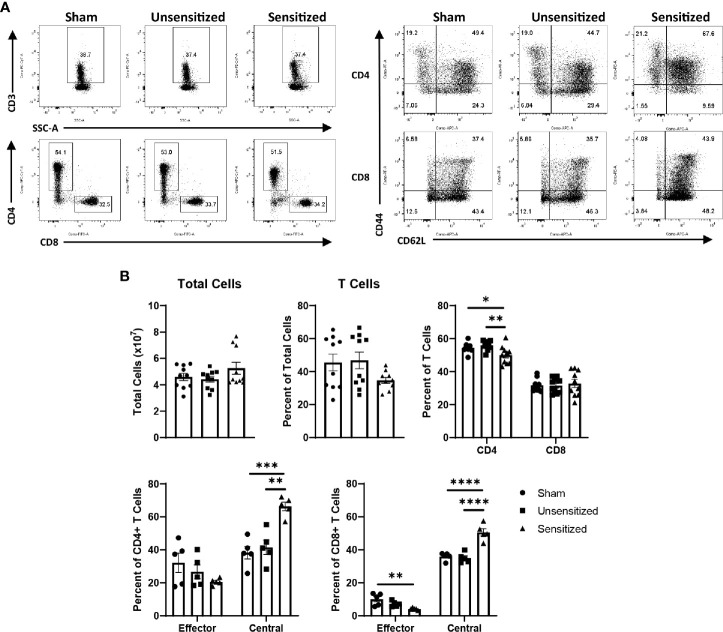
Systemic central memory T cells are expanded in sensitized mice. **(A)** Representative flow cytometry gates for CD3+ T cells, CD4+ and CD8+ T cells, and CD44+CD62L- T_EM_ and CD44+CD62+ T_CM_ of cells extracted from spleens. **(B)** Summary graphs showing the total cells extracted from spleens and the numbers of T cell populations expressed as a percentage of the parent group. Total cells extracted from spleens show no significant differences, however both sensitized mice show a mild decrease in the proportion of CD4 T cells in the spleen compared to controls. Both CD4 and CD8 T cells from sensitized mice show significant increases in T_CM_ compared to unsensitized and sham groups. CD8 T_EM_ from sensitized mice are significantly decreased compared to sham mice. Graphs show the means of each group with error bars that represent the standard error of the mean, n=5 mice per group. Data were analyzed using one-way ANOVA with Dunnett’s correction for multiple comparisons. Asterisks indicate significant differences between groups (*p < 0.05, **p < 0.01, ***p < 0.001, ****p < 0.0001).

WCV reactogenic responses reported in humans are not confined to the vaccine site and may manifest as fever, fatigue, malaise, and joint pain (9, 15). To determine if these systemic responses are reflected in systemic T cell expansion and activation, we evaluated T cell subpopulations extracted from spleens of sensitized, unsensitized, and sham mice at 14 days post-elicitation. The total numbers of cells extracted from the spleens of mice did not significantly differ between experimental groups (**Figure 8B**). Evaluation of splenocytes showed a mild, but significant decrease in the proportion of CD4+ T cells in sensitized compared to unsensitized and sham controls in response to elicitation (**Figures 8A, B**). Both CD4+ and CD8+ T cells showed significant increases in the proportions of T_CM_ compared to unsensitized and sham groups (**Figures 8A, B**). CD8+ T_EM_ from sensitized mice were decreased compared to sham but not unsensitized mice, while CD4+ T_EM_ did not differ among groups (**Figures 8A, B**).

**Figure 9:**
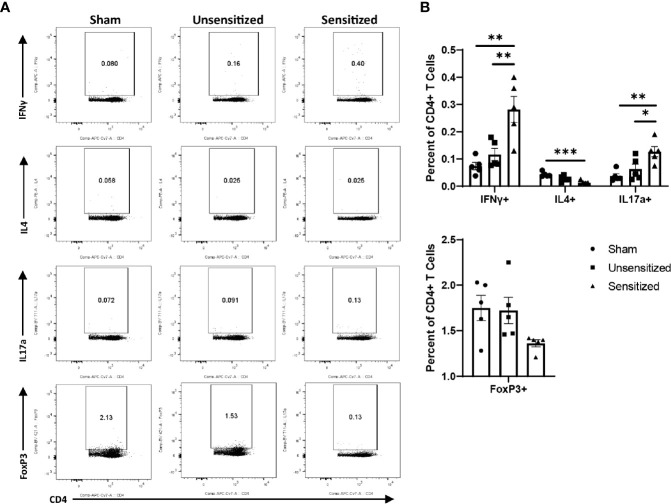
Sensitized mice show systemic increases in IFNγ+ and IL17a+ CD4 T cells. **(A)** Representative flow cytometry gates for IFNγ, IL4, IL17a, and FoxP3 expression by CD4+ T cells from spleens. **(B)** Summary graphs of IFNγ, IL4, IL17a, and FoxP3 expression. CD4+ T cells show an increase in IFNγ+ and IL17a+ cells in sensitized mice compared to unsensitized and sham groups. Sensitized mice also display reduction in IL4-expressing CD4+ T cells compared to sham mice. FoxP3+ Tregs do not significantly differ across experimental groups. Graphs show the means of each group with error bars that represent the standard error of the mean, n=5 mice per group. Data were analyzed using one-way ANOVA with Dunnett’s correction for multiple comparisons. Asterisks indicate significant differences between groups (*p < 0.05, **p < 0.01, ***p < 0.001).

Similar to local vaccine sites, splenic CD4 T cells from sensitized mice produced significantly more IFNγ+ and IL17a+ cells compared to unsensitized and sham controls. CD4 T cells from sensitized mice also showed significantly less IL4+ cells than sham mice (**Figures 9A, B**). FoxP3+ CD4 T cells did not differ across experimental groups (**Figures 9A, B**). Among CD8 T cells in the spleen, IFNγ+ cells were significantly increased in unsensitized mice compared to sensitized mice, but not sham mice. There were no significant differences in IL4+ and IL17a+ CD8 T cells among experimental groups (**Supplementary Figure 3**).

## Discussion

*C. burnetii* whole cell vaccine reactogenic responses have long stood as a barrier to the widespread availability of protective vaccines against *C. burnetii* infection (11, 12). Despite the importance of preventing adverse responses to vaccination in developing novel vaccines, little is understood about the pathophysiology of these reactions. Although guinea pigs are the current preferred animal model to evaluate reactogenicity of novel vaccine candidates, the lack of guinea pig-specific markers limits investigation of vaccine reactions in this species (11, 12, 16). To facilitate immunologic evaluation of local and systemic *C. burnetii* reactogenic responses, we tested the ability of three strains of mice: SKH1, C57Bl/6, and BALB/c, to produce reactions to WCV, with and without prior sensitization, similar to those described in humans.

SK and C57 mice produced significant local reactions after infection- and vaccine-sensitization. Despite hair removal in C57 and BAL strains, local responses were only observable grossly in SK mice making them a good model for gross and histologic evaluation of reactogenicity in novel *C. burnetii* vaccine candidates. In contrast, sensitized BAL mice did not produce more severe local reactions compared to unsensitized controls despite developing high anti-*C. burnetii* IgG titers after sensitization. Since BAL mice have a Th2-skewed immunophenotype where C57 and SK mice are Th1-skewed, this gave an early indication that *C. burnetii* WCV reactions are likely Th1-mediated (26, 27). Sensitization by either infection or immunization did not significantly alter the local lesions on histology in terms of severity or quality of the granulomatous inflammation. While all tested elicitation doses produced significantly more severe reactions in sensitized mice, the 10 µg dose provided the most significant differences in lymphocyte infiltration between sensitization groups, indicating that this would be the best dose for later evaluation of adaptive T cell responses within vaccine reaction sites. The lack of a proportionate increase in T cell responses in sensitized mice at the 30 µg dose may be due to decreased CD4 T cell responses reported with high doses of antigens compared to low doses. High doses of antigens in vaccines targeting intracellular infectious agents have been shown to induce exhaustion and terminal differentiation in T cells, tolerance among antigen-specific T cells, and apoptosis of T cells with high avidity (32). Although both SK and C57 mice readily produced significant hyper-reactive lesions to vaccination with WCV, C57 mice were chosen for subsequent experiments because of their inbred background and the wide variety of available congenic strains that will allow for future investigation of the immunologic mechanisms of *C. burnetii* WCV reactogenicity.

To investigate the pathophysiology of local and systemic *C. burnetii* reactogenic responses, T cell subpopulations isolated from the vaccine sites and spleens were evaluated by flow cytometry. Within vaccine sites, a significant increase in IFNγ+ CD4 T cells was observed in sensitized mice, indicating a Th1 type response. Canonically, Th1 cells enhance M1 macrophage polarization which increases ROS production, phagocytic activity, and induces production of cytokines that recruit neutrophils and stimulate Th1 and Th17 cells (33). On histopathology, WCV reactive lesions show infiltrations of activated macrophages, neutrophils, and lymphocytes with central areas of suppurative necrosis. Thus, the results of our evaluation of local T cell subpopulations concur with the morphology of the local lesions and infiltrating immune cells described in our experiments. Traditionally, CD4 T cells were thought to mediate cutaneous hyper-reactive responses, however, studies in contact hypersensitivity have shown that either CD4 or CD8 cells may facilitate these responses (34–36). The results of this work suggest that WCV reactogenicity is mediated by CD4 rather than CD8 T cells, however, effector CD8 T cells may cause lesions by production of molecules other than the ones assessed here, such as granzyme B and perforin (19, 37). While our data does not indicate a requirement for CD8 T cells in *C. burnetii* WCV reactogenic responses, further studies would be needed to determine if they contribute to the local or systemic responses.

Locally, we also observed increased numbers of CD4+IL17a+ Th17 cells in sensitized mice but not unsensitized or sham mice. Th17 cells have been associated with severe lesions in hyper-reactive responses as well as auto-immune diseases (38, 39). These cells produce IL17a and other cytokines that enhance neutrophil activation and recruitment (29, 40, 41). In our experiments, although neutrophils were present in vaccine sites of both sensitized and unsensitized mice, areas of suppuration and necrosis were only evident in sensitized mice. This suggests that Th17 cell activation is necessary for suppuration of neutrophils and abscess formation in *C. burnetii* WCV reactions. Additionally, Th17 cells have been associated with the formation of ELFs through their production of IL17 and IL22. ELFs are areas of inducible lymphoid tissue that form in response to chronic antigenic stimulation and correlate with severity of inflammation in some diseases (40). In our experiments, ELFs were present in both sensitized and unsensitized mice, but at the 10 µg elicitation dose, ELFs were more numerous in sensitized mice. Thus, although Th17 cells may not be necessary to form ELFs in WCV reactogenic responses, they may enhance ELF formation at lower elicitation doses.

The effect of Treg cells on cutaneous hypersensitivity responses has been investigated in several studies on contact hypersensitivity (42–44). Treg cells abrogate inflammation during elicitation of hypersensitivities by the production of IL10 and adenosine. This Treg response is considered essential for the resolution of inflammation in mouse models of contact hypersensitivity (42, 43). Our experiments showed no significant influx of CD4+FoxP3+ Treg cells in vaccine reactions and spleens of sensitized mice at 14 days post-elicitation compared to controls. This lack of Treg response during elicitation despite the late time point is likely contributing to the prolonged inflammation. This is may be at least partially due to the Th17 response evident in sensitized mice. Treg cells form when naïve CD4 T cells are activated in the presence of transforming growth factor β1 (TGFβ). However, naïve CD4 T cells activated with a combination of low levels of TGFβ and high levels pro-inflammatory cytokines, such as IL1 and IL6, will form of Th17 cells instead (45). Thus, Th17 cell activation prevents the formation of Treg cells at sites of severe inflammation. This process may explain the lack of Treg response in *C. burnetii* WCV hypersensitivity reactions.

Our experiments showed local influx of central, effector, and resident memory CD4 and CD8 T cells during WCV reactogenic responses. Memory-inducing immune responses in peripheral tissues produce not only T_CM_ and T_EM_ but tissue-specific T_RM_ as well (46–48). These memory T cell subpopulations likely develop from a common naïve T cell precursor (47). In sites of inflammation, central memory and effector memory T cells may home to ELFs that form in peripheral tissues (49). ELF formation was more frequent in sensitized mice at the 10 µg elicitation dose on histopathology and immunohistochemistry and can partially explain the local expansion of these T cell subpopulations. The increase in T_EM_ and T_RM_ suggests a role for both circulating and tissue specific memory T cell responses during elicitation of WCV reactogenic responses (47, 48). Since T_RM_ normally home to the originally affected tissue, it is reasonable to suspect that local reactions may be altered depending on the route of sensitization (30, 46). However, in our initial experiments, no differences were observed in the severity or the types of infiltrating immune cells in local reactions when comparing infection- and vaccine-sensitized mice. In experiments on contact hypersensitivity, inhibition of T_RM_ causes a delayed hyper-reactive response during elicitation compared to controls indicating that although T_RM_ produce a more rapid response to re-stimulation, T_RM_ are not necessary to induce reactive lesions in the skin (47). Interestingly, in our experiments, infection-sensitized SK mice began developing local induration two days earlier than vaccine-sensitized mice when elicited with WCV. However, infection-sensitized SK mice had higher anti-*C. burnetii* IgG titers than vaccine-sensitized mice and this may simply reflect variability in the degree of sensitization. Understanding the roles of circulating and resident memory cells in mediating *C. burnetii* WCV reactions warrants further studies.

o investigate systemic responses to WCV, we also evaluated changes in T cell populations within the spleen during elicitation. In humans, systemic reactions to *C. burnetii* vaccination are frequent and include headache, lethargy, fever, and joint pain (9, 15). In our experiments, systemic T cell responses during the elicitation phase were milder than local reactions. However the increase in IFNγ- and IL17a-producing CD4 T cells and expansion of central memory T cells in the spleens of sensitized mice indicate that *C. burnetii* WCV reactogenic responses in mice are not confined to the local vaccine site. Elevations in several circulating cytokines have been implicated in systemic reactions to vaccination including IL1β, IL6, and tumor necrosis factor α (TNFα), as well as C-reactive protein (50). Elevated IFNγ levels in serum have been correlated with systemic symptoms of reactogenicity 7 days post-vaccination with a modified live smallpox vaccine and after booster doses of a liposome adjuvanted hepatitis vaccine (51, 52). While IL1β, IL6, and TNFα are early innate mediators of inflammation, IFNγ is mainly secreted by activated T cells, which may explain the delayed onset of reactions and reactions following boost, but not prime, doses of vaccines (19, 50). Although IL17a has not been implicated in systemic reactions to vaccines, increases in systemic IL17a have been associated with disease severity in systemic auto-immune diseases in humans such as rheumatoid arthritis, systemic lupus erythematosus, and psoriasis and may be contributing to systemic adverse reactions to *C. burnetii* WCV as well (39, 41).

Here we presented a novel sensitized mouse model of *C. burnetii* WCV reactogenic responses which reproduces local vaccine reactions similar to those reported in humans. Our mouse model expands on the ability to investigate adverse reactions to *C. burnetii* whole cell vaccines and novel vaccine candidates compared to the guinea pig model because of the greater availability of immunologic markers and commercially available congenic strains which allows for evaluation of complex immune responses. Similarly, use of a vaccine-sensitized model rather than an infection-sensitized model permits experimental investigation outside of BSL-3 facilities. We demonstrated that local *C. burnetii* WCV-induced reactions in sensitized mice are characterized by an increase in IFNγ- and IL17a-producing CD4 T cells indicating a Th1-type hypersensitivity response. The similar increases in IFNγ+ and IL17a+ CD4 T cells in the spleens of sensitized mice suggest a potential pathophysiology for systemic reactogenic responses to *C. burnetii* WCV reported in humans. Our work provides insights into the pathophysiology of *C. burnetii* WCV reactogenic responses which will help guide the development of novel vaccines against *C. burnetii* that are protective without causing adverse reactions.

## Data Availability Statement

The original contributions presented in the study are included in the article/**Supplementary Material**. Further inquiries can be directed to the corresponding author.

## Ethics Statement

The animal study was reviewed and approved by Institute of Animal Care and Use Committee at Texas A&M University.

## Author Contributions

Experiments were designed by AF, AG, ES, and JS. AF performed experiments, analyzed data, and wrote the manuscript. AG performed experiments and prepared the vaccine material. All authors contributed to the article and approved the submitted version.

## Funding

This research was supported by the Wofford Cain Endowed Research Fund and the Defense Threat Reduction Agency, contract HDTRA1-14-C-0113. Additional support was provided by the National Institutes of Health Institutional Training Grant T32 fellowship 5 OD 11083-11.

## Conflict of Interest

The authors declare that the research was conducted in the absence of any commercial or financial relationships that could be construed as a potential conflict of interest.

## Publisher’s Note

All claims expressed in this article are solely those of the authors and do not necessarily represent those of their affiliated organizations, or those of the publisher, the editors and the reviewers. Any product that may be evaluated in this article, or claim that may be made by its manufacturer, is not guaranteed or endorsed by the publisher.

## Supplementary Material

Supplemental Figure 1 Post-elicitation monitoring for weight change and vaccine site induration. **(A)** Presence of local induration in each experimental group. Only infection- and vaccine- sensitized SK mice showed grossly visible induration at the elicitation site. **(B)** Change in weight during 14 days post-elicitation. There is no significant weight loss in any experimental group during elicitation. Weight data shows the means of each group (n=5) with error bars showing the standard error of the mean.

Supplemental Figure 2 CD8 T cells from vaccine sites do not show increased production of IFNγ, IL4, or IL17a. **(A)** Representative gates for IFNγ, IL4, and IL17a production by CD8+ T cells from vaccination sites. **(B)** Summary of total IFNγ+, IL4+, and IL17a+ CD8+ T cells. There were no significant differences in cytokine production by CD8+ T cells across experimental groups. Graphs show the means of each group with error bars that represent the standard error of the mean. Cell counts are the sum of four vaccination sites from each mouse, n=5 mice per group. Data were analyzed using one-way ANOVA with Dunnett’s correction for multiple comparisons.

Supplemental Figure 3 Flow cytometric evaluation of cytokine production by CD8+ T cells extracted from spleens. **(A)** Representative flow cytometry gates for IFNγ, IL4, and IL17a production by CD8+ T cells from spleens. **(B)** Summary of total IFNγ+, IL4+, and IL17a+ CD8+ T cells. IFNγ+ CD8+ T cells are mildly increased compared to sensitized mice but not sham mice. There are no significant changes in IL4+ and IL17a+ CD8+ T cells across groups. Graphs show the means of each group with error bars that represent the standard error of the mean. Cell counts are the sum of four vaccination sites from each mouse, n=5 mice per group. Data were analyzed using one-way ANOVA with Dunnett’s correction for multiple comparisons. Asterisks indicate significant differences between groups (*p < 0.05).
