# Supplementary figures and images for: Coxiella burnetii Whole Cell Vaccine Produces a Th1 Delayed-Type Hypersensitivity Response in a Novel Sensitized Mouse Model
Source: Front Immunol. 2021 Sep 20;12:754712. doi: 10.3389/fimmu.2021.754712 (PMC8488435; doi:10.3389/fimmu.2021.754712)

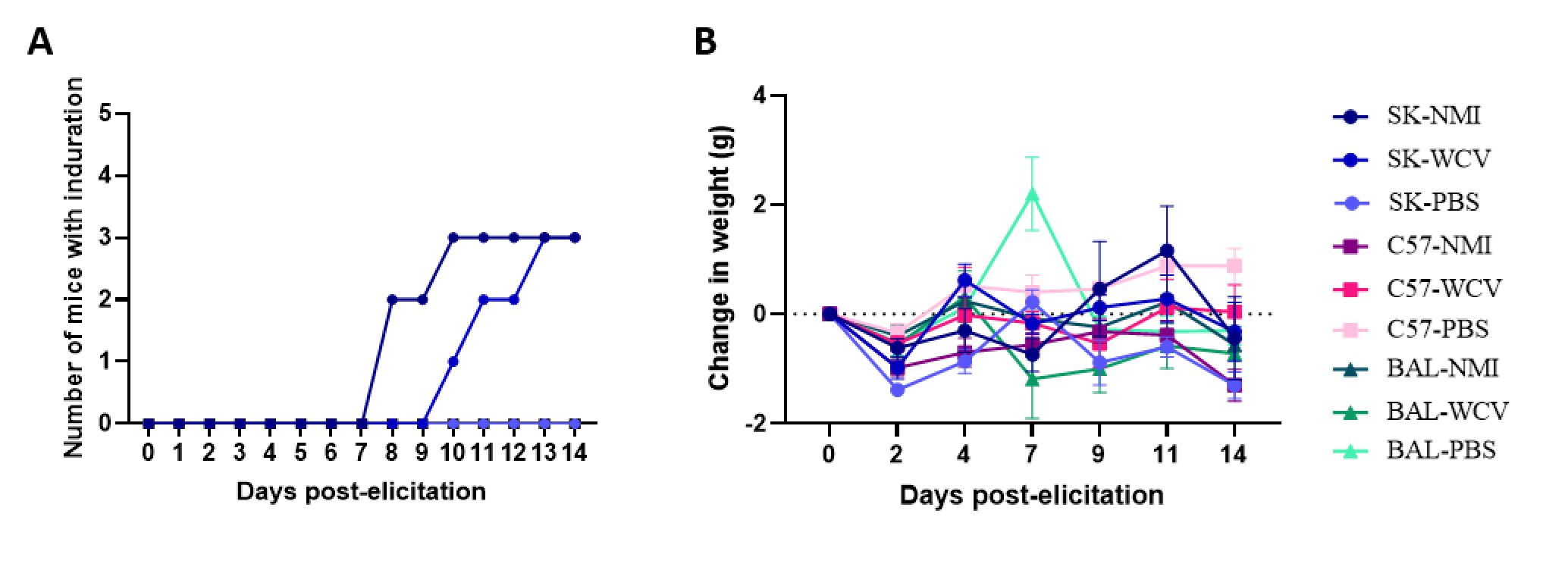

Supplement: Supplemental Figure 1 — Post-elicitation monitoring for weight change and vaccine site induration. (A) Presence of local induration in each experimental group. Only infection- and vaccine- sensitized SK mice showed grossly visible induration at the elicitation site. (B) Change in weight during 14 days post-elicitation. There is no significant weight loss in any experimental group during elicitation. Weight data shows the means of each group (n=5) with error bars showing the standard error of the mean. [file Image_1.tif]

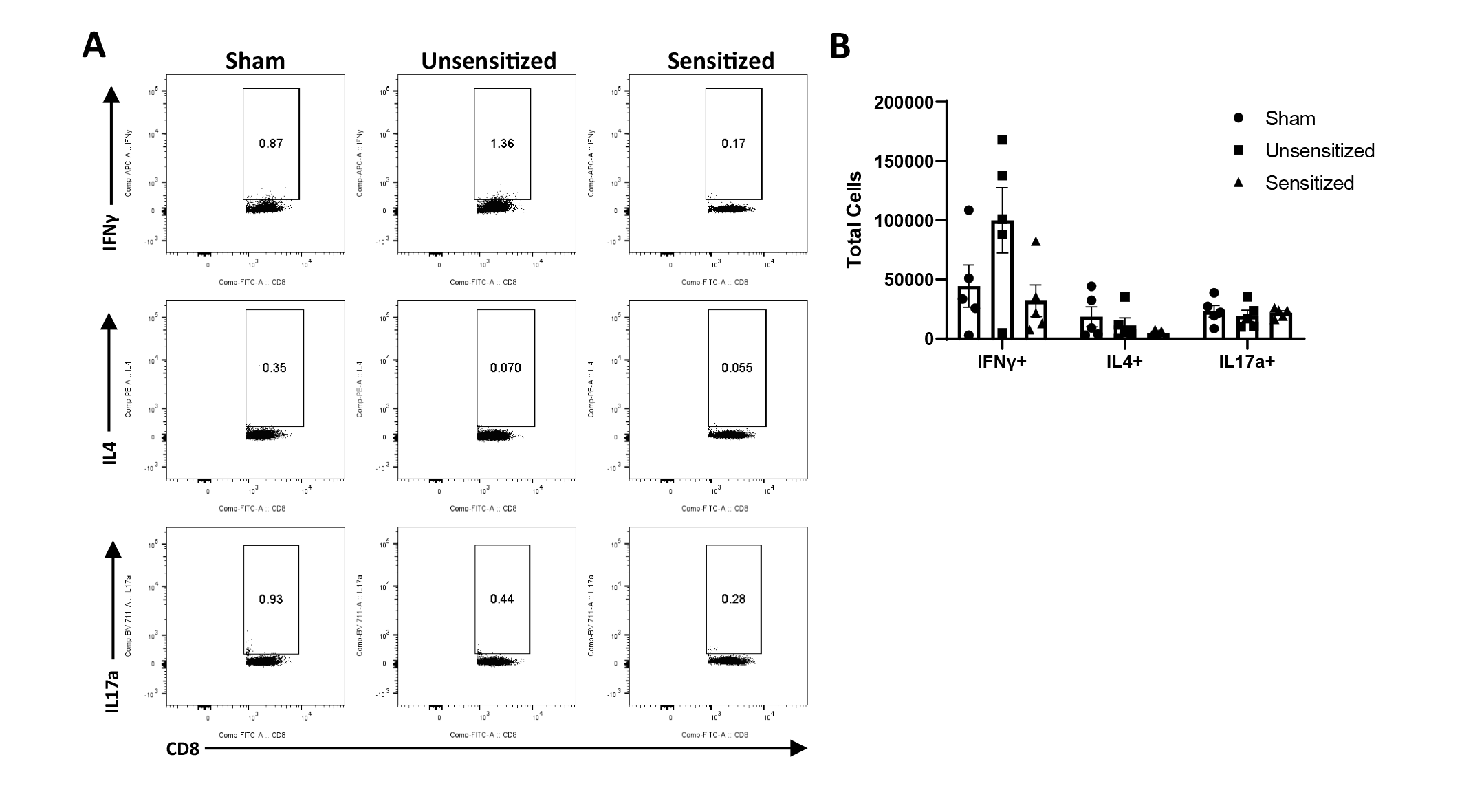

Supplement: Supplemental Figure 2 — CD8 T cells from vaccine sites do not show increased production of IFNγ, IL4, or IL17a. (A) Representative gates for IFNγ, IL4, and IL17a production by CD8+ T cells from vaccination sites. (B) Summary of total IFNγ+, IL4+, and IL17a+ CD8+ T cells. There were no significant differences in cytokine production by CD8+ T cells across experimental groups. Graphs show the means of each group with error bars that represent the standard error of the mean. Cell counts are the sum of four vaccination sites from each mouse, n=5 mice per group. Data were analyzed using one-way ANOVA with Dunnett’s correction for multiple comparisons. [file Image_2.tif]

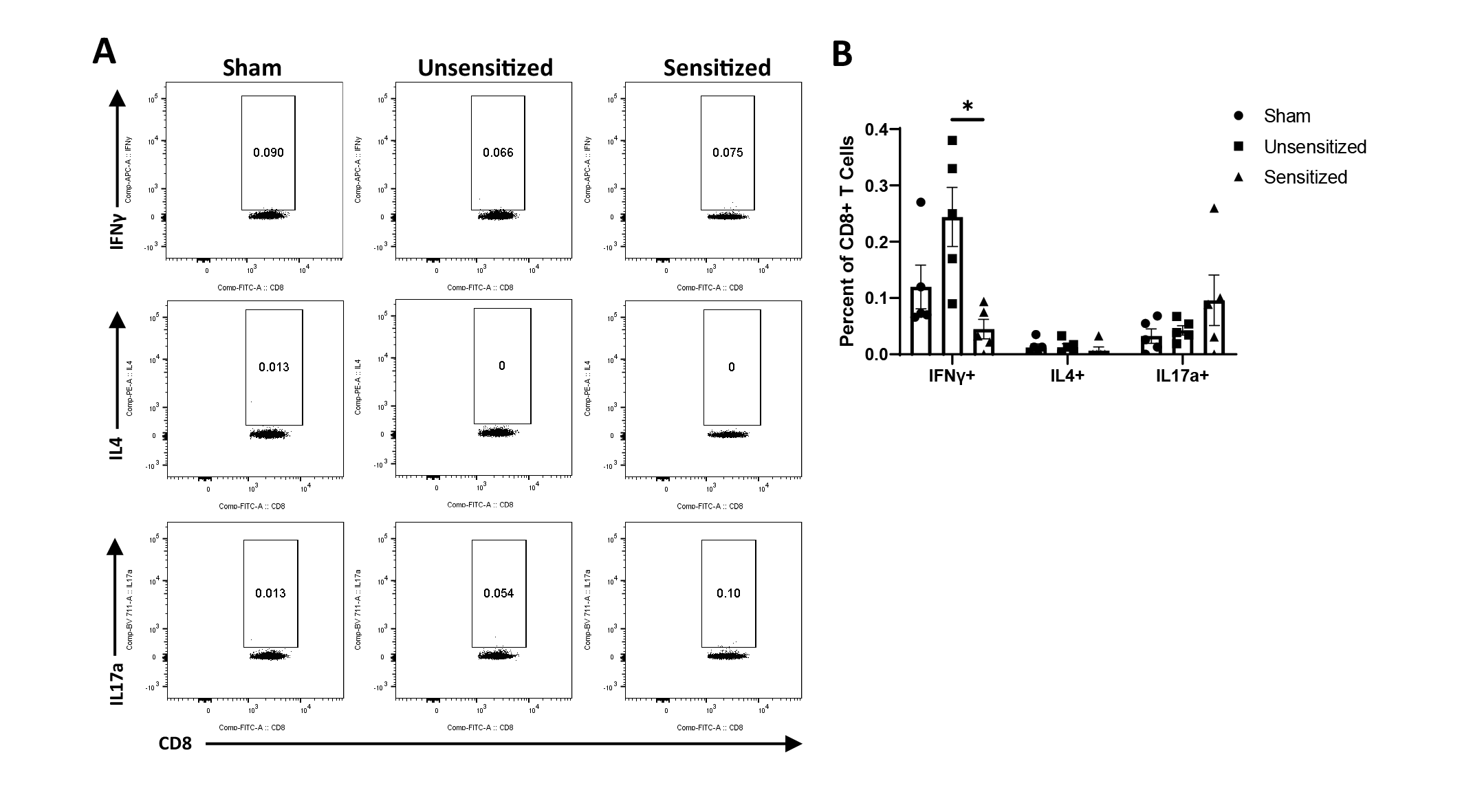

Supplement: Supplemental Figure 3 — Flow cytometric evaluation of cytokine production by CD8+ T cells extracted from spleens. (A) Representative flow cytometry gates for IFNγ, IL4, and IL17a production by CD8+ T cells from spleens. (B) Summary of total IFNγ+, IL4+, and IL17a+ CD8+ T cells. IFNγ+ CD8+ T cells are mildly increased compared to sensitized mice but not sham mice. There are no significant changes in IL4+ and IL17a+ CD8+ T cells across groups. Graphs show the means of each group with error bars that represent the standard error of the mean. Cell counts are the sum of four vaccination sites from each mouse, n=5 mice per group. Data were analyzed using one-way ANOVA with Dunnett’s correction for multiple comparisons. Asterisks indicate significant differences between groups (*p < 0.05). [file Image_3.tif]
